# Probable Microcystin Toxicosis in a Red-Gartered Coot (*Fulica armillata*) from a Protected Coastal Wetland in Central Chile—A Sentinel for Toxic Cyanobacterial Bloom?

**DOI:** 10.3390/vetsci13060508

**Published:** 2026-05-23

**Authors:** Juliana Souza, Luis Araya, Maria Elisa Vergara, Raquel Pinto, Beatriz Escobar, André V. Rubio, Antonella Bacigalupo, Christian Hidalgo, Diego Ramírez-Alvarez, Claudia Foerster, Morgane Derrien, Gemma Rojo

**Affiliations:** 1 FaunaLab, Instituto de Ciencias Agroalimentarias, Animales y Ambientales (ICA3), Universidad de O’Higgins, San Fernando 3070000, Chile; juliana.souza@pregrado.uoh.cl (J.S.); luis.araya@ac.uoh.cl (L.A.); maria.vergara@ac.uoh.cl (M.E.V.); 2 Departamento de Medicina Preventiva Animal, Facultad de Ciencias Veterinarias y Pecuarias, Universidad de Chile, Santiago 8820808, Chile; raquel.pinto@uoh.cl (R.P.); beatrizescobar@uchile.cl (B.E.); 3 Departamento de Ciencias Biológicas Animales, Facultad de Ciencias Veterinarias y Pecuarias, Universidad de Chile, Santiago 8820808, Chile; arubio@uchile.cl; 4 Centro de Modelamiento y Perspectiva One Health (CEMPOH), Facultad de Medicina Veterinaria y Agronomía, Universidad de las Américas, Santiago 8370065, Chile; abacigalupo@udla.cl (A.B.); chidalgo@udla.cl (C.H.); 5 Unidad de Vida Silvestre, Servicio Agrícola y Ganadero Región de O’Higgins, Rancagua 2841051, Chile; diego.ramirez@sag.gob.cl; 6 Laboratorio de Inocuidad Alimentaria (LIA), Instituto de Ciencias Agroalimentarias, Animales y Ambientales (ICA3), Universidad de O’Higgins, Ruta 90 km 3, San Fernando 3070000, Chile; claudia.foerster@uoh.cl; 7 BioGeoQuím Lab, Instituto de Ciencias Agroalimentarias, Animales y Ambientales (ICA3), Universidad de O’Higgins, San Fernando 3070000, Chile; morgane.derrien@uoh.cl

**Keywords:** Red-gartered coot, *Fulica armillata*, microcystin, cyanobacterial bloom, *Microcystis aeruginosa*, wildlife pathology, sentinel species, One Health, wetland, Chile

## Abstract

Cyanobacterial harmful algal blooms are an increasing concern for wildlife health, particularly in eutrophic wetlands, yet well-documented avian cases supported by environmental, pathological, and toxicological evidence remain scarce. This study describes a sentinel case of probable microcystin toxicosis in a Red-gartered coot (*Fulica armillata*) from Laguna Petrel, a protected coastal wetland in central Chile, during a broader wildlife mortality event. Surface-water monitoring included nutrient analyses, in situ physicochemical measurements, phytoplankton assessment, and cyanotoxin quantification. The evaluated bird was documented alive with severe motor impairment, euthanized, and examined by gross necropsy, histopathology, and tissue toxicology. Water analyses showed elevated nutrients, persistently alkaline and highly productive conditions, marked dominance of *Microcystis aeruginosa*, and high concentrations of microcystin-LR, microcystin-RR, microcystin-YR, and nodularin. The bird showed marked hepatic lesions at necropsy, histopathological changes compatible with acute hepatotoxic injury, and detectable microcystin-LR in lyophilized liver tissue. Taken together, these findings support a diagnosis of probable microcystin toxicosis in this individual. This case highlights the value of waterfowl as sentinels of ecosystem health threats and underscores the importance of integrated monitoring in protected coastal wetlands potentially affected by toxic cyanobacterial blooms.

## Simple Summary

Toxic blooms caused by cyanobacteria are becoming a growing problem in wetlands and can threaten ecosystem health, including wildlife and people. In this study, we investigated the case of a Red-gartered coot found affected in Laguna Petrel, a protected coastal wetland in central Chile, during a period when the water showed signs of physicochemical quality deterioration. Our goal was to determine whether the bird had been affected by toxins produced during a bloom of these microorganisms. Water analyses showed high nutrient levels, conditions favoring excessive growth, and very high concentrations of harmful toxins. The bloom was dominated by *Microcystis aeruginosa*, a species well known for producing liver-damaging toxins. The bird showed severe movement issues before being euthanized. After death, examination of the body showed marked liver damage; microscopic analysis confirmed severe injury to liver cells, and toxin analysis detected microcystin in liver tissue. Together, these findings support that the bird was probably affected by cyanobacterial toxins. This study shows how wild waterfowl can act as early warning signs of environmental health problems and highlights the need for better monitoring and protection of coastal wetlands that are important for biodiversity and public well-being.

## 1. Introduction

Cyanobacterial harmful algal blooms are increasingly recognized as a major concern for ecosystem health, including wildlife and people [1]. These blooms are commonly associated with nutrient enrichment, eutrophication, elevated pH, and hydrological conditions that favor cyanobacterial proliferation [2]. Among cyanotoxins, microcystins are especially relevant because they are potent hepatotoxins capable of affecting domestic animals, wildlife, and humans [2,3].

One of the best-known bloom-forming cyanobacteria is *Microcystis aeruginosa*, an important producer of hepatotoxic microcystins [4]. The species has been recognized as a dominant component of toxic blooms in eutrophic waters, and its toxins are associated with illness and mortality in animals exposed through contaminated water or biomass [5,6]. Microcystin toxicity is mainly linked to inhibition of protein phosphatases 1 and 2A, which leads to disruption of hepatocellular architecture and acute liver injury [4,7]. In shallow wetlands and coastal lagoons, wildlife may be especially vulnerable because exposure can occur through drinking, feeding, and direct contact with contaminated water [8].

Birds are particularly useful as sentinels of environmental deterioration because they are visible, ecologically linked to aquatic habitats, and often respond quickly to local hazards [8]. Within a One Health framework, sentinel wildlife can provide early warning of toxic blooms and their broader implications for ecosystem integrity [9]. Waterfowl are tightly connected to wetland functioning, and can reflect local environmental deterioration through changes in behavior, morbidity, and mortality, because of contaminant exposure [8]. Even so, proving cyanotoxin involvement in wildlife morbidity or mortality is often difficult. In many cases, carcasses are found too late, tissue sampling is incomplete, or environmental evidence is insufficient to support a strong diagnosis [1].

Several reports have linked cyanobacteria and their toxins with avian mortality risk. Waterbird mass mortality has been documented in Doñana National Park, Spain [10], and toxic cyanobacteria have also been considered in mortality events affecting flamingos in alkaline Rift Valley lakes, eastern Africa [11]. These studies support the broader idea that aquatic birds may be exposed to harmful blooms either directly through water ingestion or indirectly through trophic pathways in highly productive wetlands [10,11]. Nevertheless, individual bird cases supported simultaneously by bloom characterization, tissue toxin detection, and compatible pathology remain relatively uncommon.

The Red-gartered coot (*Fulica armillata*) is a strongly wetland-associated waterbird, commonly linked to shallow aquatic habitats and vegetation in coastal systems of Chile and southern South America [12,13]. This close ecological relationship with near-surface water and aquatic vegetation supports its interpretation as a plausible sentinel organism. More broadly, waterfowl are recognized as useful sentinels of environmental hazards because they are visible, ecologically connected to aquatic food webs, and often respond rapidly to toxic bloom exposure [8].

Laguna Petrel is a protected coastal wetland in central Chile. Like other coastal wetlands in central Chile, it exists within a geomorphologically dynamic landscape influenced by marine processes and regional coastal deformation [14,15]. Coastal wetlands in Chile are increasingly recognized as environmentally valuable but vulnerable systems, especially where urbanization, hydrological alteration, and broader climate-related pressures affect ecological function [16]. In 2025, Laguna Petrel presented eutrophic conditions followed by a toxic cyanobacterial bloom dominated by *Microcystis aeruginosa*. During the same period, a broader wildlife mortality event was recorded, including two *Myocastor coypus*, two *Xenopus laevis,* and several gulls. One Red-gartered coot (*Fulica armillata*) was recovered alive with severe motor impairment and became the only bird from this event for which postmortem examination, histopathology, and tissue toxicology could be performed. The aim of this study was to characterize the environmental scenario and clinicopathological findings of this sentinel case in Laguna Petrel, and to explore whether the combined evidence supports a diagnosis of probable microcystin toxicosis in wild waterfowl.

## 2. Materials and Methods

### 2.1. Study Area and Environmental Context

This study was conducted in Laguna Petrel, Pichilemu, central Chile (34°22′58.60″ S, 71°59′54.50″ W). The wetland is a protected shallow coastal system subject to seasonal hydrological variation and episodic marine influence. During 2025, field observations documented visible eutrophic conditions, followed by a broader wildlife mortality event affecting multiple species. Only one bird was recovered in a condition suitable for complete postmortem examination, histopathology, and tissue toxicology.

### 2.2. Environmental Sampling Design

**Figure 1:**
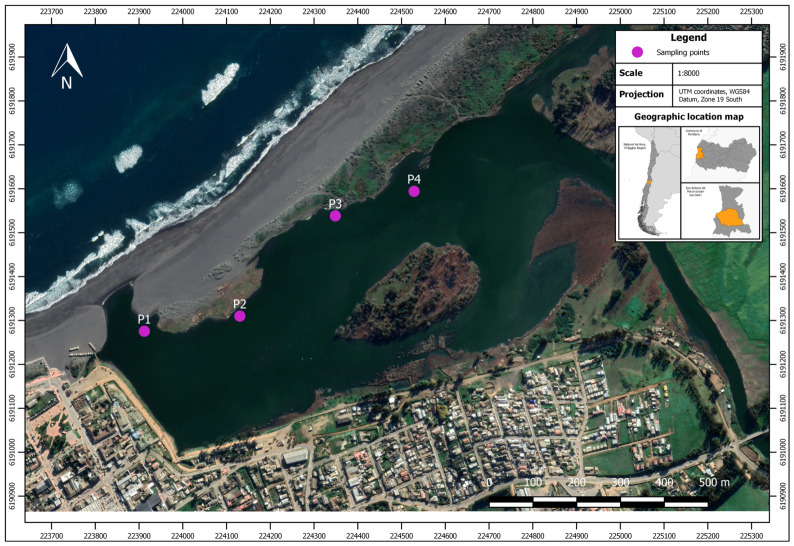
Satellite view of Laguna Petrel showing the sampling points (PE1–PE4) distributed along the lagoon’s coastal margin and their proximity to the urban area of Pichilemu.

Environmental monitoring was conducted at four fixed sampling stations within Laguna Petrel (PE1, PE2, PE3, and PE4) between 24 April and 10 June 2025. The sampling design aimed to capture spatial variability within the wetland by repeatedly surveying the same locations (Figure 1).

In situ physicochemical measurements were performed at all stations on three dates: 24 April, 29 May, and 10 June 2025.

Surface-water samples were collected on selected dates for specific analyses. On 24 April 2025, samples were collected for nutrient characterization, while on 10 June 2025, a surface-water sample was collected for phytoplankton and cyanotoxin analyses.

Therefore, the environmental component of this study should be interpreted as an event-linked descriptive characterization of bloom-favorable conditions, rather than as a spatially replicated or temporally continuous assessment of bloom dynamics throughout the entire lagoon.

### 2.3. In Situ Physicochemical Measurements

In situ measurements were recorded at each sampling station using a multiparameter water quality instrument (Hanna Instruments, HI98194, Villafranca Padovana, Italy). The recorded variables included pH, dissolved oxygen concentration, electrical conductivity, total dissolved solids, salinity, and temperature. Measurements were taken directly in the field at PE1–PE4 on each sampling date following the manufacturer’s operating procedures for the instrument used.

### 2.4. Water Chemistry

Surface-water samples collected on 24 April 2025 were analyzed for total phosphorus, nitrate, nitrite, total Kjeldahl nitrogen, total nitrogen, pH, conductivity, temperature, turbidity, and total organic carbon by an external laboratory. According to the laboratory report, total Kjeldahl nitrogen corresponded to an accredited analysis.

### 2.5. Phytoplankton Analysis

Surface-water samples collected on 10 June 2025 were analyzed for phytoplankton composition and abundance by an external laboratory. Quantitative phytoplankton microscopy was performed using an inverted microscope and a sedimentation-based counting procedure consistent with the Utermöhl method [17]. Taxonomic identification and cell counts were reported by the laboratory, and the dominant cyanobacterial taxon was identified as *Microcystis aeruginosa*, with abundance expressed as cells per liter.

### 2.6. Water Cyanotoxin Analysis

The same 10 June 2025 water sample was analyzed for cyanotoxins by an external laboratory. According to the analytical report, microcystins and nodularin were determined using solid phase extraction and high-performance liquid chromatography (HPLC) with ultraviolet detection following ISO 20179:2005. The quantified analytes included microcystin-LR, microcystin-RR, microcystin-YR, nodularin, and microcystin-LA. Results were reported in micrograms per liter.

### 2.7. Sentinel Bird Case and Clinical Documentation

A Red-gartered coot (*Fulica armillata*) was first detected in Laguna Petrel on 23 June 2025 through field observation and video footage. According to the field record, the bird was unable to rise properly and showed marked motor impairment and abnormal head movements. The bird was euthanized on 24 June 2025 due to its clinical condition. Avian influenza testing was performed by the local animal health authority (SAG) using real-time RT-PCR on tracheal and cloacal swabs, targeting the influenza A matrix gene. The result was negative, as documented in Supplementary File S1.

The bird was the only individual from the broader wildlife event recovered in adequate condition for full examination. Additional carcasses associated with the event were found in an advanced state of decomposition and could not be subjected to comparable diagnostic procedures.

### 2.8. Necropsy

The gross necropsy was performed after euthanasia. The examination included external inspection and systematic evaluation of the oral cavity, thoracic cavity, abdominal cavity, liver, heart, air sacs, gastrointestinal tract, serosal surfaces, and pectoral musculature. Gross findings were recorded descriptively. Attention was given to lesions compatible with acute toxic injury, including hepatic enlargement, congestion, hemorrhage, and abnormal discoloration.

### 2.9. Histopathology

Representative tissue samples were collected during necropsy and processed for histopathology according to routine diagnostic procedures. Tissues were fixed in All-Fix (Cancer Diagnostics, Durham, NC, USA), embedded in paraffin, sectioned, stained with hematoxylin and eosin, and examined by light microscopy. Histopathological interpretation focused on liver and heart lesions. The liver was evaluated for hepatocellular degeneration, vascular congestion, inflammatory infiltrates, and cholestatic change. Myocardial sections were evaluated for edema and degenerative changes.

### 2.10. Tissue Cyanotoxin Analysis

Liver tissue from the Red-gartered coot was collected during necropsy using clean instruments and placed in a sterile polypropylene tube. The sample was maintained frozen at −20 °C from collection until shipment and was transported to the analytical laboratory under frozen conditions. Upon receipt, the sample was immediately processed. The liver sample was lyophilized, processed by methanolic extraction, and analyzed by high-performance liquid chromatography with diode-array detection. The final cyanotoxin concentration was expressed in mg/kg of lyophilized tissue. The quantified analytes included microcystin-LR, microcystin-RR, microcystin-YR, nodularin, and microcystin-LA.

### 2.11. Data Handling and Interpretation

All data were analyzed descriptively. Nutrient concentrations, field physicochemical measurements, phytoplankton abundance, cyanotoxin concentrations in water, and pathological findings were organized by date and sampling point to reconstruct the environmental context of the event. Because this study reports a sentinel case within a broader mortality episode and not a controlled experiment, no inferential statistical analyses were performed.

Environmental, pathological, and toxicological findings were interpreted jointly to assess whether the evidence supported probable microcystin toxicosis in the evaluated bird.

### 2.12. Ethical Approval

This study involved the evaluation of a free-ranging wild bird recovered during a wildlife health event. Euthanasia, necropsy, and sample collection were performed in the context of wildlife rescue and diagnostic investigation, with approval by the local animal health authority.

### 2.13. Use of Generative AI

Generative AI was used in the creation of the graphical abstract.

## 3. Results

### 3.1. Environmental Conditions Before the Bird Mortality Event

In situ physicochemical measurements recorded between April and June 2025 showed that Laguna Petrel remained consistently alkaline and presented conditions compatible with a eutrophic, photosynthetically active system. In this context, productivity was inferred from the combination of elevated nutrient concentrations, high pH, increased dissolved oxygen and oxygen saturation during daytime measurements, and the later detection of dense cyanobacterial biomass dominated by *Microcystis aeruginosa* (Table 1). On 24 April, pH ranged from 8.81 to 8.91, and dissolved oxygen from 6.86 to 9.98 mg/L. By 29 May, pH had increased to 9.17–9.33, dissolved oxygen to 10.05–12.91 mg/L, and oxygen saturation to 109.4–139.1%. On 10 June, pH remained high (9.22–9.33), dissolved oxygen ranged from 11.51 to 13.90 mg/L, and oxygen saturation increased again, from 112.8 to 141.3%.

Conductivity and salinity changed markedly among sampling dates. Conductivity increased from 14,420–14,610 µS/cm in April to 24,350–25,200 µS/cm in late May, then decreased to 13,010–13,200 µS/cm on 10 June. Salinity showed a similar pattern, increasing from 8.40–8.53 PSU in April to 14.85–15.41 PSU in May and then decreasing to 7.53–7.64 PSU in June. These data indicate that Laguna Petrel combined eutrophic conditions with substantial short-term hydrological variability.

Water chemistry analyses performed on 24 April 2025 showed elevated nutrient concentrations at all four sampling stations in Laguna Petrel (Table 2). Total phosphorus ranged from 1.8 to 2.1 mg/L, nitrite from 0.28 to 0.56 mg/L, and total nitrogen from 3.18 to 3.84 mg/L. Water pH was alkaline at all points (8.52–8.73), and laboratory conductivity values ranged from 15,229 to 15,426 µS/cm, indicating brackish conditions.

### 3.2. Bloom Characterization and Cyanotoxin Detection in Water

Phytoplankton analysis of the 10 June 2025 surface-water sample showed marked dominance of *Microcystis aeruginosa*, with an abundance of 113,770,800 cells/L. Cyanotoxin analysis of the same water sample detected multiple hepatotoxic cyanobacterial compounds (Table 3). These results demonstrate that the bloom was not only dominated by a known toxin-producing cyanobacterium but was also associated with high concentrations of cyanotoxins in surface water.

### 3.3. Clinical Presentation of the Sentinel Bird

A Red-gartered coot (*Fulica armillata*) was first documented alive in Laguna Petrel on 23 June 2025. According to field observation and video records (Supplementary File S2), the bird showed severe motor impairment, inability to rise properly, and abnormal head movements (Table 4).

Avian influenza testing performed on the same day was reported as negative in the field case record. This result reduced support for a major influenza-associated explanation in the evaluated bird, although it did not exclude all alternative diagnoses.

### 3.4. Gross Pathological Findings

Gross necropsy identified the liver as the main affected organ. The organ was enlarged and showed severe congestion, multifocal hemorrhagic areas, and darkened regions interpreted as compatible with acute hepatocellular injury. These lesions were the most prominent pathological findings in the bird.

Other body systems showed fewer relevant changes. Serosal surfaces were moist but lacked fibrinous or purulent exudate. Pectoral musculature was preserved, with no evidence of marked emaciation. No gross abnormalities were observed in the air sacs, intestines, crop, proventriculus, or gizzard, and no foreign bodies were detected. The heart was proportionate in size and showed no gross evidence of pericarditis or hemopericardium. In the oral cavity, the tongue showed focal discoloration and darkening at the base and dorsal surface, interpreted cautiously as possible vascular compromise or terminal circulatory change.

Overall, gross examination indicated a lesion pattern dominated by acute hepatic injury rather than generalized traumatic, obstructive, or suppurative disease.

### 3.5. Histopathological Findings

The main lesions were moderate-to-severe vacuolar degeneration of hepatocytes, marked sinusoidal and portal vascular congestion, mild inflammatory infiltrate, and mild cholestatic change. This pattern was interpreted as highly compatible with acute hepatotoxic injury.

**Figure 2:**
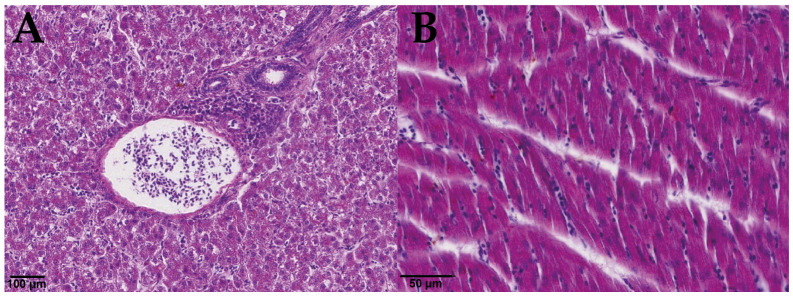
Histopathological findings in the sentinel Red-gartered coot (*Fulica armillata*): (**A**) liver showing acute hepatotoxic injury; (**B**) heart showing mild secondary myocardial changes. Hematoxylin and eosin. Size bar: (**A**) 100 µm; (**B**) 50 µm.

Myocardial tissue preserved its overall architecture but showed mild interstitial edema and early degenerative change. Compared with the liver lesions, cardiac alterations were less pronounced and were interpreted as compatible with secondary systemic involvement rather than a primary cardiac process (Figure 2).

### 3.6. Cyanotoxin Detection in Liver Tissue

Toxicological analysis of lyophilized liver tissue detected microcystin-LR at 60.24 mg/kg (Table 4). Nodularin, microcystin-RR, microcystin-YR, and microcystin-LA were not detected in the analyzed sample.

The detection of microcystin-LR in liver tissue provides direct evidence that the bird had internalized a cyanobacterial hepatotoxin present in the wetland during the bloom period, strengthening the diagnosis of probable microcystin toxicosis.

### 3.7. Integrated Interpretation of the Sentinel Case

Taken together, the results document a coherent sequence linking environmental deterioration and toxic bloom conditions with the pathological and toxicological findings in the sentinel bird. Before the bird mortality event, Laguna Petrel showed elevated nutrient concentrations, persistent alkalinity, and high oxygen saturation, all consistent with a highly productive eutrophic system. In June, the wetland presented a dense *Microcystis aeruginosa* bloom and high concentrations of cyanotoxins in surface water. Within this context, the Red-gartered coot showed severe clinical impairment, marked hepatic lesions at necropsy, histopathological changes consistent with acute hepatotoxic injury, and detectable microcystin-LR in liver tissue.

These results support a diagnosis of probable microcystin toxicosis in the evaluated bird. However, because equivalent diagnostic evaluation was not possible in the additional decomposed carcasses, the findings support inference at the level of the sentinel case rather than definitive attribution of the entire broader wildlife mortality event.

## 4. Discussion

This study describes a sentinel case of probable microcystin toxicosis in a Red-gartered coot from Laguna Petrel, identified during a broader wildlife mortality event in a protected coastal wetland. The case is strengthened by the convergence of environmental, clinical, pathological, and toxicological evidence.

The wetland environment was already at risk of a cyanobacterial bloom before the bird was detected. Cyanobacterial blooms are widely associated with eutrophication, nutrient loading, alkaline conditions, and hydrological instability [2]. In Laguna Petrel, elevated nutrient concentrations were documented in April, followed by persistently alkaline and highly productive physicochemical conditions through May and June. By 10 June, the bloom had been analytically characterized and was clearly dominated by *Microcystis aeruginosa*, one of the most widely recognized bloom-forming and microcystin-producing cyanobacteria [4]. Earlier work has also long recognized *M. aeruginosa* as a dominant cyanobacterium in toxic blooms from eutrophic waters, reinforcing the biological plausibility of this environmental scenario [5,6]. However, because phytoplankton composition and cyanotoxin concentrations were obtained from a single surface-water sample, our data cannot resolve fine-scale spatial heterogeneity or temporal bloom dynamics across the whole lagoon. The repeated measurements at PE1–PE4 provide useful physicochemical context, but the taxonomic and cyanotoxin results represent a point-in-time characterization during the event period. Accordingly, we avoid extrapolating these findings to the entire wetland as a continuous ecological process and interpret them as evidence that toxic bloom conditions were present in Laguna Petrel during the period in which wildlife morbidity and mortality were observed.

The coot presented severe motor impairment before euthanasia. Although these signs were not specific, the recent literature has emphasized that paretic syndromes in wild birds require broad differential diagnosis, including infectious, toxic, nutritional, and contaminant-related causes [18]. In this case, avian influenza testing was reportedly negative, and the bird came from a wetland where a toxic cyanobacterial bloom had already been confirmed.

Microcystins are primarily hepatotoxins, and their biological activity is linked to inhibition of protein phosphatases 1 and 2A, leading to the disruption of hepatocellular cytoskeletal integrity and acute liver damage [4,7]. This mechanism fits well with the lesions observed in the present case. Gross necropsy revealed hepatomegaly, severe congestion, multifocal hemorrhage, and dark hepatic areas, while histopathology showed moderate-to-severe hepatocellular vacuolar degeneration, vascular congestion, and mild cholestatic change. Importantly, microcystin-LR was also detected in liver tissue, substantially strengthening the interpretation that the bird experienced biologically relevant toxin exposure. Similar reasoning has been applied in previous studies that documented microcystin contamination in tissues of exposed vertebrates from eutrophic systems with toxic *Microcystis* blooms, including birds and other aquatic wildlife [19,20].

The detection of nodularin in the water sample also requires cautious interpretation. Although the phytoplankton report identified *Microcystis aeruginosa* as the dominant cyanobacterium, the presence of nodularin suggests that the toxin profile may not be fully explained by the dominant taxon alone [21]. One possibility is that a low-abundance or spatially heterogeneous co-occurring cyanobacterium contributed to the toxin mixture but was not represented in the dominant-taxon summary [22]. Another possibility is that the single surface-water sample did not capture the full taxonomic and toxin variability of the bloom [23]. Therefore, we do not attribute nodularin production to *M. aeruginosa* in this case. Instead, the combined toxin profile further supports the need for more detailed cyanobacterial community characterization, including repeated sampling and, when possible, molecular or toxin-gene-based approaches in future monitoring.

At the same time, the broader wildlife mortality event must be interpreted cautiously. Additional animals associated with the event were found in advanced decomposition and could not undergo comparable postmortem or laboratory analyses. For that reason, our results do not justify assigning the full multispecies event exclusively to microcystins, since bloom-associated mortality in wild birds may involve additional or co-occurring factors, including other cyanotoxins such as neurotoxins [18], avian botulism [24], heavy metals and pesticides [25], and indirect bloom effects such as oxygen depletion or broader hydrological stress [24]. Instead, the Red-gartered coot should be understood as a sentinel case within a more complex ecological disturbance. This type of caution is important in bloom-associated mortality events, where full attribution at the event level is often limited by incomplete carcass recovery, rapid decomposition, or lack of equivalent sampling across affected species. A similar need for caution has been noted in other bloom-affected protected ecosystems, where cyanotoxin exposure co-occurs with broader environmental instability and not all affected fauna can be evaluated diagnostically [26].

This case is also relevant because Laguna Petrel is not an isolated inland waterbody, but a coastal wetland embedded in a dynamic physical setting influenced by marine processes and regional coastal change [14,15]. Such coastal dynamics may shape salinity, water exchange, and ecosystem instability, and they should be kept in mind when interpreting bloom-associated events in systems like this. More broadly, Chilean coastal wetlands are increasingly recognized as vulnerable socioecological systems, particularly under pressures related to hydrological alteration, urbanization, and climate-driven coastal hazards [16]. In addition, the repeated detection of microcystins in the wetland during the following autumn suggests that the conditions favoring these toxic events may persist or recur over time, reinforcing the need for sustained environmental monitoring and integrated surveillance within a One Health framework.

This case reinforces the need for targeted wildlife surveillance, because the detection of a clinically affected waterfowl during a cyanobacterial bloom can serve as an operational warning signal for an ongoing environmental health hazard [8]. In practical terms, such a finding should trigger rapid field actions, including systematic search and early recovery of fresh carcasses, standardized necropsy and tissue collection before decomposition, repeat water sampling for cyanotoxins, phytoplankton characterization, communication with environmental and animal health authorities, and precautionary risk communication for people, domestic animals, and wildlife using the wetland [18]. In the present case, liver cyanotoxin results were available one month after sample submission, which means that tissue toxicology primarily provided diagnostic confirmation and retrospective support for the sentinel case rather than serving as the sole basis for an immediate response. This limitation highlights the need to combine wildlife surveillance with faster field-based or laboratory water screening during suspected bloom events [27].

A key limitation of this study is the temporal separation between environmental toxin measurement and clinical documentation of the bird. Nutrient characterization was performed on 24 April 2025, phytoplankton and cyanotoxin analyses were based on a surface-water sample collected on 10 June 2025, and the Red-gartered coot was documented and examined on 23–24 June 2025. Therefore, no environmental cyanotoxin measurement was available from the exact day of illness or from the immediately preceding days. The detection of microcystin-LR in liver tissue confirms that the bird had internalized a cyanobacterial hepatotoxin, and the environmental data demonstrates that a toxic bloom was present during the same event period. However, the precise toxin concentration to which the bird was exposed at the time of illness cannot be reconstructed.

Even with its limitations, the case remains relevant from both veterinary and One Health perspectives. Cyanobacterial toxins are not only a wildlife problem; they are also relevant to domestic animals and humans sharing affected freshwater environments through drinking, feeding, and recreational or occupational exposure pathways [28]. Moreover, reports that combine environmental toxin confirmation, tissue detection, and compatible pathology in an individual waterfowl are still relatively uncommon. Therefore, this case contributes useful evidence from South America and highlights the need for integrated surveillance in coastal wetlands, including environmental monitoring, wildlife rescue, pathology, and toxin analysis.

## 5. Conclusions

Laguna Petrel showed eutrophic, bloom-favorable conditions during both the period preceding and accompanying a broader wildlife mortality event. A sentinel Red-gartered coot recovered during this period showed compatible clinical signs, marked hepatic lesions, histopathological findings consistent with acute hepatotoxic injury, and detectable microcystin-LR in liver tissue. Taken together, these findings support probable microcystin toxicosis in this bird. Because comparable diagnostic investigation was not possible in the other affected animals, the present study does not establish microcystins as the definitive cause of the entire multispecies mortality event. Instead, it highlights the veterinary and environmental relevance of affected waterfowl as sentinels and reinforces the need for integrated surveillance in protected coastal wetlands.

## Figures and Tables

**Table 1:** In situ physicochemical parameters recorded at four stations in Laguna Petrel.

Date	Sampling Point	pH	Dissolved O_2_ (mg/L)	Conductivity (µS/cm)	TDS (mg/L)	Salinity (PSU)	Temperature (°C)
24 April 2025	PE1	8.91	9.07	14,420	7211	8.40	17.54
	PE2	8.87	9.98	14,430	7216	8.41	17.08
	PE3	8.87	7.49	14,490	7245	8.45	16.94
	PE4	8.81	6.86	14,610	7307	8.53	17.52
29 May 2025	PE1	9.17	10.55	24,350	12,170	14.85	13.58
	PE2	9.22	10.05	25,200	12,600	15.41	13.67
	PE3	9.26	10.60	25,090	12,540	15.34	13.28
	PE4	9.33	12.91	24,800	12,400	15.14	13.23
10 June 2025	PE1	9.31	12.69	13,200	6601	7.64	11.93
	PE2	9.33	13.90	13,010	6508	7.53	13.22
	PE3	9.22	11.51	13,110	6560	7.59	11.42
	PE4	9.27	13.12	13,040	6522	7.54	11.89

TDS = total dissolved solids; PSU = Practical Salinity Unit.

**Table 2:** Nutrient and laboratory water chemistry data from Laguna Petrel on the four sampling points.

Sampling Point	Total Phosphorus (mg/L)	Nitrate (mg/L)	Nitrite (mg/L)	Total Kjeldahl Nitrogen ^1^ (mg/L)	Total Nitrogen ^2^ (mg/L)	pH	Conductivity (µS/cm)	Temperature (°C)	Turbidity (NTU)	Total Organic Carbon (mg/L)
PE1	2.1	0.3	0.28	2.6	3.18	8.73	15,229	21.6	5.4	20.1
PE2	2.0	0.7	0.44	2.7	3.84	8.52	15,239	21.7	12.0	17.3
PE3	1.8	0.4	0.51	2.3	3.21	8.54	15,285	21.5	7.9	36.8
PE4	2.1	0.5	0.56	2.2	3.26	8.54	15,426	21.5	12.0	21.3

^1^ Total Kjeldahl nitrogen was reported as an accredited laboratory analysis. ^2^ Total nitrogen was calculated as total Kjeldahl nitrogen + nitrate + nitrite using laboratory-reported values.

**Table 3:** Phytoplankton and cyanotoxin findings in surface water from Laguna Petrel on 10 June 2025.

Variable	Result
Dominant cyanobacterium	*Microcystis aeruginosa*
*Microcystis aeruginosa* abundance	113,770,800 cells/L
Microcystin-LR	1761.5 µg/L
Microcystin-RR	1312.1 µg/L
Microcystin-YR	276.4 µg/L
Nodularin	24.7 µg/L
Microcystin-LA	<1.0 µg/L

**Table 4:** Clinical, pathological, and toxicological findings in the sentinel Red-gartered coot (*Fulica armillata*).

Category	Finding
Species	Red-gartered coot (*Fulica armillata*)
Place of detection	Laguna Petrel, Pichilemu, central Chile
First field observation	23 June 2025
Main clinical signs	Unable to rise properly, severe motor impairment, abnormal head movements
Euthanasia date	24 June 2025
Avian influenza	Negative, according to field case record
Gross liver findings	Hepatomegaly, severe congestion, multifocal hemorrhage, dark areas compatible with acute hepatocellular injury
Other necropsy findings	No major gross lesions in air sacs, intestines, crop, proventriculus, gizzard, or pericardial sac; preserved pectoral musculature
Oral finding	Discoloration and focal darkening of the tongue, interpreted cautiously as possible vascular compromise or terminal circulatory change
Histopathology	Moderate to severe vacuolar hepatocellular degeneration, sinusoidal and portal congestion, mild inflammatory infiltrate, mild cholestatic change
Myocardial histology	Mild interstitial edema and early degenerative change
Microcystin-LR in liver tissue	60.24 mg/kg

## Data Availability

The original contributions presented in this study are included in the article/Supplementary Material. Further inquiries can be directed to the corresponding author.

## Supplementary Materials

The following supporting information can be downloaded at https://www.mdpi.com/article/10.3390/vetsci13060508/s1: Supplementary File S1: Local animal health authority certificate of negative avian influenza diagnosis; Supplementary File S2: Video recording of the affected Red-gartered coot, showing clear signs of neurological impairment.

## Abbreviations

The following abbreviations are used in this manuscript:

MC	Microcystin
MC-LR	Microcystin-LR
MC-RR	Microcystin-RR
MC-YR	Microcystin-YR
MC-LA	Microcystin-LA
PSU	Practical Salinity Unit
TDS	Total Dissolved Solids
NTU	Nephelometric Turbidity Unit
HPLC-DAD	High-Performance Liquid Chromatography with Diode-Array Detection
ISO	International Organization for Standardization
SAG	Servicio Agrícola y Ganadero
