# Supplementary material for: Probable Microcystin Toxicosis in a Red-Gartered Coot (Fulica armillata) from a Protected Coastal Wetland in Central Chile—A Sentinel for Toxic Cyanobacterial Bloom?
Source: Vet Sci. 2026 May 23;13(6):508. doi: 10.3390/vetsci13060508 (PMC13308339; doi:10.3390/vetsci13060508)
Supplement: Supplementary file 1 [file vetsci-13-00508-s001.zip › Supplementary File 1.pdf]

SAG
Ministerio de Agricultura
Gobierno de Chile

Unidad de Vida Silvestre
Departamento de Recursos Naturales Renovables
SAG Región de O'Higgins

CASE CERTIFICATE

To whom it may concern.

Through this document, based on our responsibilities for the protection of wildlife, as administrators and enforcers of Law 19473 and its implementing regulations, Decree No. 5/1998, of the Ministry of Agriculture of the Republic of Chile, I hereby declare that:

Within the framework of our wildlife health surveillance activities, on June 24, 2025, in the Laguna Petrel Protected Wetland, Pichilemu, O'Higgins Region, a *Fulica armillata* individual was found exhibiting symptoms consistent with an acute neurological condition. The animal was sampled for our Avian Influenza surveillance program, with a subsequent negative result. Given its clinical presentation, our preventative health system, and the need to safeguard animal welfare, the individual was euthanized on site, and its carcass was delivered, with appropriate biosafety measures, for subsequent analysis, to Professor Gemma Rojo of the Faunalab Laboratory, University of O'Higgins.

Issued in Rancagua, on August 20, 2025.

SERVICIO AGRICOLA Y GANADERO
VIDA SILVESTRE
REGION DE O'HIGGINS

Diego Ramírez Alvarez. MV. MSc.
Coordinador Regional Unidad de Vida Silvestre
Servicio Agrícola y Ganadero SAG
Región de O'Higgins
